# Propensity score analysis of 18-FDG PET/CT-enhanced staging in patients undergoing surgery for esophageal cancer

**DOI:** 10.1007/s00259-018-4118-9

**Published:** 2018-08-16

**Authors:** N. Patel, Kieran G. Foley, A. G. Powell, J. R. Wheat, D. Chan, P. Fielding, S. A. Roberts, W. G. Lewis

**Affiliations:** 1 Department of General Surgery, University Hospital of Wales, Cardiff, CF14 4XW UK; 2 Division of Cancer & Genetics, Cardiff University School of Medicine, Heath Park, Cardiff, CF14 4XN UK; 3 Wales Research & Diagnostic Positron Emission Tomography Imaging Centre (PETIC), UHW, Cardiff, CF14 4XN UK; 4 Department of Radiology, University Hospital of Wales, Cardiff, CF14 4XW UK

**Keywords:** Esophagus, Neoplasms, Positron emission tomography, Survival, Recurrence

## Abstract

**Purpose:**

PET/CT is now integral to the staging pathway for potentially curable esophageal cancer (EC), primarily to identify distant metastases undetected by computed tomography. The aim of this study was to analyze the effect of PET/CT introduction on survival and assess patterns of recurrence after esophagectomy.

**Methods:**

A longitudinal cohort of EC patients staged between 1998 and 2016 were considered for inclusion. After co-variate adjustment using propensity scoring, a cohort of 496 patients (273 pre-PET/CT and 223 post-PET/CT) who underwent esophagectomy [median age 63 years (31–80), 395 males, 425 adenocarcinomas, 71 squamous cell carcinomas, 325 neoadjuvant therapy] were included. The primary outcome measure was overall survival (OS) based on intention to treat.

**Results:**

Three-year OS pre-PET/CT was 42.5% compared with 57.8% post-PET/CT (Chi^2^ 6.571, df 1, *p* = 0.004). On multivariable analysis, pT stage (HR 1.496 [95% CI 1.28–1.75], *p* < 0.0001), pN stage (HR 1.114 [95% CI 1.04–1.19], *p* = 0.001) and PET/CT staging (HR 0.688 [95% CI 0.53–0.89] *p* = 0.004) were independently associated with OS. Recurrent cancer was observed in 125 patients (51.4%) pre-PET/CT, compared with 74 patients post-PET/CT (37.8%, *p* = 0.004), and was less likely to be distant recurrence after PET/CT introduction (39.5 vs. 27.0%, *p* = 0.006).

**Conclusions:**

Enhanced PET/CT staging is an important modality and independent factor associated with improved survival in patients undergoing esophagectomy for cancer.

## Introduction

Imaging is fundamental to improved cancer staging and largely guides treatment decision-making. Radiological staging investigations including computed tomography (CT) and positron emission tomography (PET) provide anatomical and functional information and have greatly impacted clinical practice [1–3].

Positron emission tomography integrated with computed tomography (PET/CT) is now an established and evidence-based part of the modern radiological staging algorithm of esophageal cancer (EC) [4, 5]. Reported benefits include the detection of distant metastases not detected by CT, which changes management in up to 38% of patients [6].

Yet PET/CT is not without limitations. Endoscopic ultrasound (EUS) has been reported to be superior in staging both the primary tumor and local lymph nodes [7], and no evidence has yet emerged that use of PET/CT has been associated with improved overall survival (OS) [8]. Moreover, important reconfiguration of UK esophagogastric cancer services has occurred over the last decade, which has been accompanied by better clinical outcomes. The National Oesophago-Gastric Cancer Audit (NOGCA) has emphasized that better patient selection by improved radiological and physiological staging accuracy, more use of neo-adjuvant chemotherapy, less post-operative morbidity and mortality, and centralization of services are all significant factors in improving survival after potentially curative esophagectomy [9]. Determining the particular role PET/CT may have played in influencing outcome after potentially curative esophageal cancer surgery is consequently challenging, and likely not to undergo a randomized control trial process.

Propensity score (PS) analysis facilitates measurement of the probability of receiving a particular treatment modality related to a number of variables, and represents a strong and powerful alternative strategy to explore cause, compared with orthodox statistical adjustment [10]. PS analysis adjusts for potential confounders, balancing important co-variables such as baseline characteristics or therapies, and is an alternative to control trials where ethical or practical challenges prohibit randomization, enhancing fair comparison [11, 12].

The aim of this study was to analyze the influence of 18-FDG PET/CT introduction on OS after esophagectomy for cancer when compared with historical controls by means of PS analysis. The hypothesis was that PET/CT introduction into the routine staging algorithm of patients diagnosed with EC was associated with improved OS after potentially curative surgery. The setting was a UK Upper Gastrointestinal (UGI) cancer network serving a population of 1.8 million.

## Materials and methods

All patients diagnosed with EC of any cell type who underwent surgery and had PET/CT imaging during the preoperative staging period in the South East Wales regional UGI cancer network were studied prospectively between January 1, 2009 and August 31, 2016. These patients were compared with a historical cohort of consecutive patients undergoing EC surgery between January 1, 1998 and January 1, 2009, staged with the network’s historical staging algorithm pre-PET/CT. Exclusion criteria included patients undergoing PET/CT for Siewert type III esophagogastric junctional cancer with proximal esophageal extension, and patients undergoing salvage esophagectomy following initial definitive chemoradiotherapy (dCRT).

**Fig. 1:**
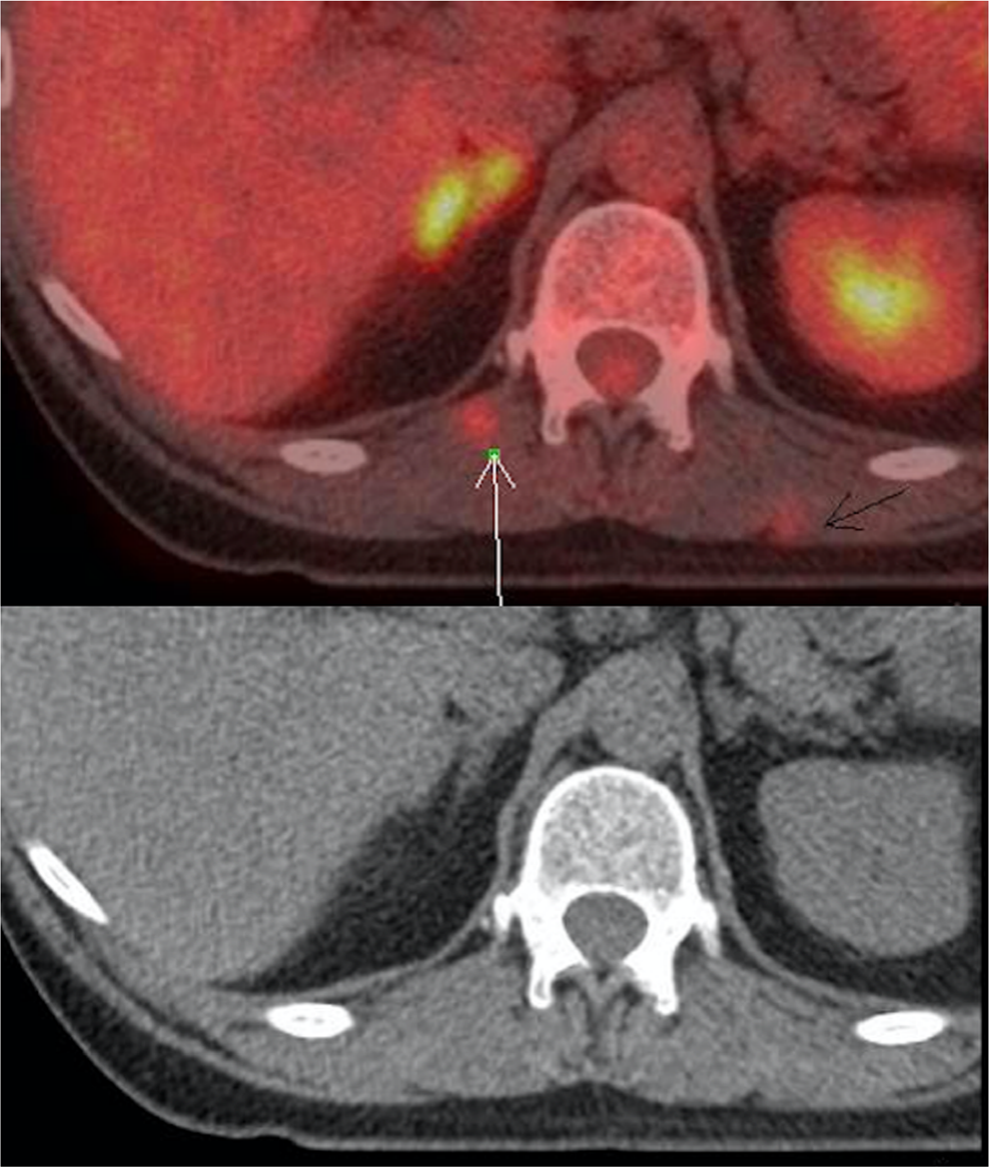
Fused PET/CT (*above*) and CT (*below*) images of soft tissue metastases (*white* and *black arrows*) in a patient initially considered suitable for curative treatment. However, the management became palliative once the metastases were detected

Patients proceeded to PET/CT staging only if they were suitable for potentially curative treatment on the grounds of CT stage and performance status, and was concurrently arranged with EUS examination. PET/CT was used for pre-treatment staging only (Fig. 1) and was not used for restaging after neoadjuvant therapy. All PET studies were integrated PET/CT and no patients received PET imaging alone. Detail of the networks’ EUS staging protocol has been described previously [13]. Patients’ fitness was assessed by cardiopulmonary exercise testing (CPX) [14], and the final management plan was determined at the regional cancer network multi-disciplinary team (MDT) meeting. All staging investigations were reported in accordance with the UICC Tumour Node Metastasis (TNM) staging methods [15]. The primary outcome measure was OS from diagnosis. Secondary outcome measures were proven recurrence patterns and disease-free survival (DFS). Ethical approval, sought from the regional ethics committee, was waived because the study was deemed to represent service evaluation. A number of developments in the management of EC occurred during the study period, including changes in practice based upon the publication of randomized clinical trials, the introduction of an enhanced recovery program in 2008 [14] and finally, centralization of the Upper Gastrointestinal (UGI) cancer regional network service in South East Wales from August 1, 2010 [16].

### PET/CT protocol

PET/CT examinations were performed at two centers. At the first center (site 1), a total of 87 patients had PET/CT examinations performed using a Philips 16 slice Gemini GXL dedicated PET/CT scanner (Philips Medical Systems, Cleveland, OH, USA). The uptake time was 60 min. A standard administered activity of 350 MBq of FDG was given. Reconstructions were performed using a 3D acquisition with non-time of flight acquisition for 4 min per bed position.

Four hundred and eighty-five patients were imaged at the second center (site 2) using a GE Discovery 690 PET/CT scanner (GE Healthcare, Pollards Wood, Buckinghamshire, UK). Serum glucose levels were routinely checked and confirmed to be less than 7.0 mmol/l prior to imaging. Patients received an activity of 4 MBq of ^18^F-FDG per kilogram of body weight. Uptake time was 90 min. PET images were acquired at 3 min per field of view. The length of the axial field of view was 15.7 cm. Images were reconstructed with the ordered subset expectation maximization algorithm, with 24 subsets and two iterations. Matrix size was 256 × 256 pixels, using the VUE Point ™ time of flight algorithm. CT images were acquired in a helical acquisition with a pitch of 0.98 and a tube rotation speed of 0.5 s. Tube output was 120 kVp with output modulation between 20 and 200 mA. Matrix size for the CT acquisition was 512 × 512 pixels with a 50-cm field of view. At both centers, all patients were starved for a minimum of 6 h prior to imaging and no oral or intravenous contrast was administered.

The assignment of lymph nodes as involved or uninvolved on PET images at the two centers was based on subjective clinical assessment in each case. In general, nodes were only assigned as involved if they showed discernible tracer uptake above that of background and were identified separately from the primary tumor. Other factors affecting the classification of nodes were the morphology, size, and relative uptake of the node in question in comparison to the primary tumor. No specific SUV or size cut off was used in assignment of nodes as benign or malignant. Lymph nodes considered physiological or related to an alternative etiology were excluded from the N stage.

### Treatment

Patients were selected for radical treatment (surgery or dCRT) based on perceived radiologic stage, comorbidity, and patient choice according to algorithms described previously [17–19]. The standard surgical approach consisted of subtotal trans-thoracic esophagectomy (TTO) as described by Lewis and Tanner. Trans-hiatal esophagectomy (THO), as described by Orringer, was used selectively in patients with adenocarcinoma of the lower third of the esophagus with significant cardiorespiratory risk profiles, or T1/2 N0 disease. All procedures used an open approach, and esophageal resection was defined as potentially curative if all visible tumor was removed, and both proximal and distal resection margins were free of tumor on histological examination. R1 resection was defined as positive longitudinal or circumferential margin status on histological examination [20].

### Follow-up evaluation

Patients were reviewed every 3 months for the first year, and 6 monthly thereafter until 5 years or death. Patients underwent clinical assessment and venous blood sampling including carcinoembryonic antigen (CEA) measurements. Patients who were suspected to have disease recurrence based on clinical assessment or a raised venous CEA underwent computed tomography or endoscopy. Patterns of recurrence were defined as loco-regional, distant (metastatic), or both loco-regional and distant, when both were diagnosed concurrently. The time of recurrence was taken as the date of the confirmatory investigation. The patient cohort was analyzed in January 2017. No patients were lost to follow-up and death certification was obtained from the Office for National Statistics via Cancer Network Information System Cymru (CaNISC).

### Statistical methods

Grouped data were expressed as median (range) and non-parametric methods used throughout. Propensity scores were generated using appropriate logistic regression model, and included all relevant independent variables thought to be potential confounding factors and those which affect outcome. Co-variate adjustment was performed using the propensity scores, which were assessed for balance across groups using a Student’s *t* test and defined by |d| > 0.25. The variables were considered by the MDT and comprised age group, gender, tumor histology, pathological T and N stage, operation type, and site of PET/CT examination. The dependent variable was whether the patient underwent PET/CT staging. The probabilities option was chosen to generate propensity scores, which were then used in a regression, covariate adjustment, to estimate the effect of exposure of PET/CT on DFS and OS. DFS was calculated by measuring the interval from a landmark time of 6 months after diagnosis to the date of recurrence; an approach mirroring previous randomized trials [21, 22] and allowing for variance in time to definitive surgery. Events resulting in a failure to complete curative treatment, such as palliative surgery, operative mortality, and disease progression during neoadjuvant therapy, were assumed to occur at this landmark time, to facilitate intention-to-treat analysis. OS was measured from the date of diagnosis, and cumulative survival calculated according to the method of Kaplan and Meier. Differences between groups were analyzed with the log rank test. Univariable analyses examining factors influencing survival were examined initially by the life table method of Kaplan and Meier, and variables significant at the *p* < 0·010 level were entered into a forward conditional Cox proportional hazards model. All statistical analysis was performed with SPSS® (IBM® SPSS® Statistics v23.0.0.0, IBM Corporation, Armonk, NY, USA).

## Results

**Fig. 2:**
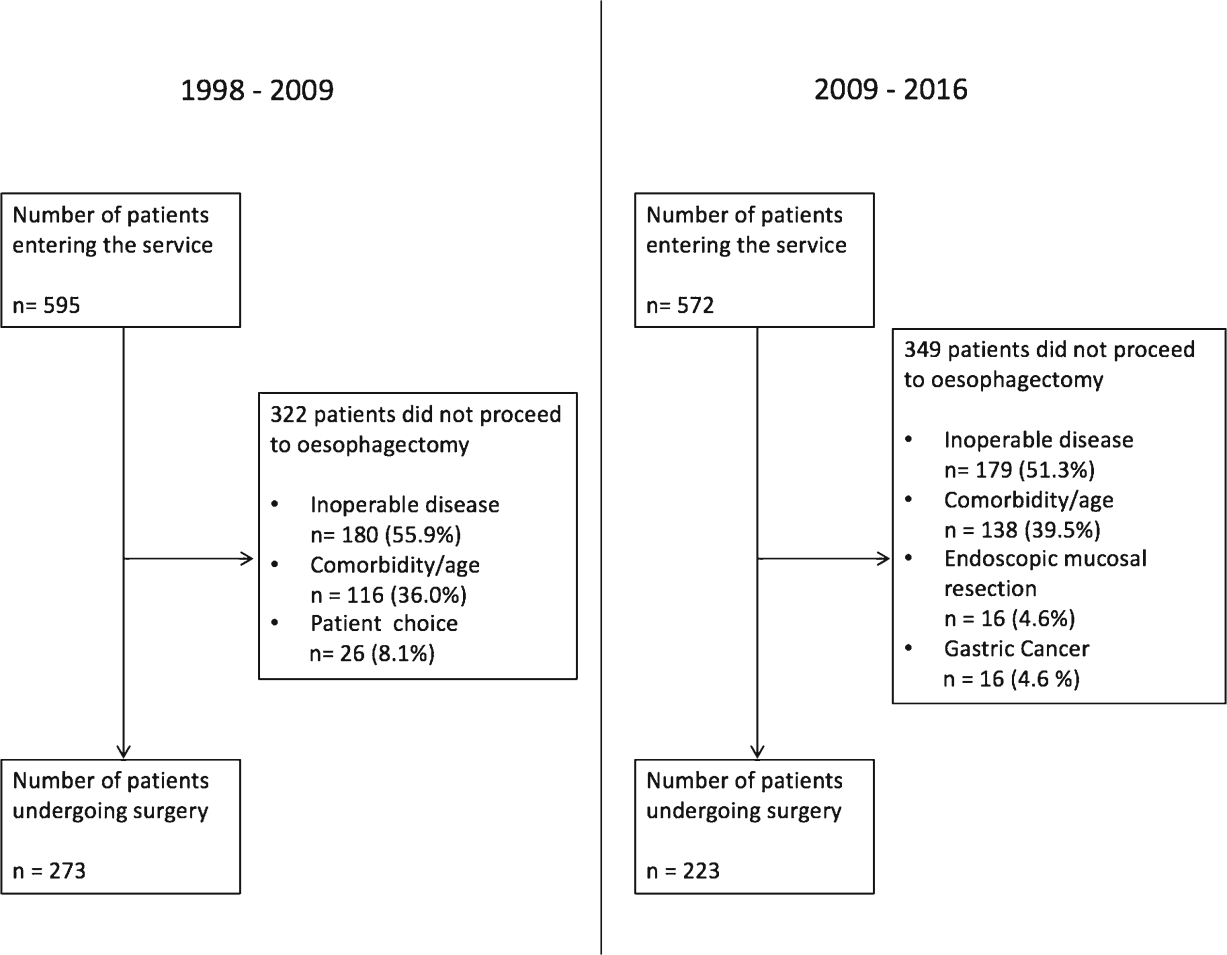
CONSORT diagram detailing the creation of both pre-PET and post-PET cohorts

In total, 1167 patients were diagnosed with EC between 1998 and 2016 (Fig. 2). Of these, 595 were diagnosed between 1998 and 2009 (pre-PET cohort) and 572 were diagnosed between 2009 and 2016 (post-PET cohort). Of the 595 in the pre-PET cohort, 322 (54.1%) did not proceed to resection because 180 (55.9%) had inoperable disease, 116 (36.0%) had pre-existing comorbidity, and 26 (8.1%) because of patient choice. Following the introduction of PET/CT (2009–2016), 572 patients were diagnosed with esophageal and junctional cancers of which 223 (40.0%) underwent EC resection. Of the 349 (61.0%) patients that did not progress to surgical resection, 179 (51.3%) had inoperable disease and 138 (39.5%) had pre-existing co-morbidity precluding curative treatment. Sixteen (4.6%) had predominantly gastric cancer extending above the esophagogastric junction (an exclusion criterion in this study), and 16 (4.6%) underwent endoscopic mucosal resection (EMR).

Following propensity scoring, 496 consecutive patients were eligible for inclusion, 273 pre-PET/CT, and 223 patients post-PET/CT. Twenty-two (9.9%) patients had non-avid tumors on PET/CT, but were included on an intention-to-treat analysis basis. Propensity scores were generated for all 496 patients in the cohort. The propensity scores were balanced across the two groups, Student’s *t* test *p* = 0.212 and there was no covariate exhibiting a large imbalance (|d| > 0.25). Patient demographics are detailed in Table 1.

**Table 1:** Details of the patients before and after PET/CT introduction

	Pre-PET/CT	Post-PET/CT	*p* value
Number (%)	273 (55.0)	223 (45.0)	
Median age years (range)	61 (31–80)	64 (36–77)	**0.036§**
< 50	38 (13.9)	23 (10.3)	
50–59	80 (29.3)	51 (22.9)	
60–69	100 (36.6)	97 (43.5)	
> 70	55 (20.2)	52 (23.3)	
Gender			0.323†
Male	213 (78.0)	182 (81.6)	
Female	60 (22.0)	41 (18.4)	
Tumor type			**0.011†**
Adenocarcinoma	224 (82.1)	201 (90.1)	
SCC	49 (17.9)	22 (9.9)	
Oncological therapy			
Neoadjuvant – all types	176 (66.4)	149 (66.8)	0.584†
Neoadjuvant chemoRx	126 (71.5)	109 (73.1)	0.493†
Neoadjuvant CRTx	50 (28.4)	40 (26.8)	0.914†
Surgery alone	97 (35.6)	74 (33.2)	0.584†
Operation type			**< 0.0001**†
TTO	150 (54.9)	83 (37.2)	
THO	93 (34.1)	107 (50.0)	
3 Stage	0 (0)	6 (2.7)	
Open and close	30 (11.0)	27 (12.1)	
Pathological T stage			**0.012†**
HGD/CPR	16 (5.8)	17 (7.6)	
T1	33 (12.1)	48 (21.5)	
T2	31 (11.4)	24 (10.8)	
T3	141 (51.6)	101 (45.3)	
T4	22 (8.1)	6 (2.7)	
Open and close	30 (11.0)	27 (12.1)	
Pathological N stage			0.381†
N0	112 (41.0)	103 (46.2)	
N1	67 (24.5)	48 (21.5)	
N2	38 (13.9)	33 (14.8)	
N3	26 (9.5)	12 (5.4)	
Open and close	30 (11.0)	27 (12.1)	
Pathological Stage (TNM 7)			0.069†
CPR	15 (5.0)	13 (5.8)	
Stage I	48 (17.6)	56 (25.1)	
Stage II	60 (22.0)	48 (19.7)	
Stage III	20 (44.0)	75 (33.6)	
Stage IV	0 (0)	2 (0.8)	
R1 resection	83 (30.4)	83 (37.2)	0.198†
Operative mortality	11 (4.0)	6 (2.7)	0.415†

*ACA* adenocarcinoma, *SCC* squamous cell carcinoma, *neoadjuvant therapy* –all types, chemotherapy/chemo radiotherapy; *neoadjuvant chemoRx*, neoadjuvant chemotherapy, *neoadjuvant CRTx*, neoadjuvant chemo radiotherapy, *THO* trans hiatal esophagectomy, *TTO* transthoracic esophagectomy; *3 stage* 3-stage esophagectomy; *HGD/CPR* high-grade dysplasia/complete pathological response, *R1* positive resection margin, *§* Mann–Whitney *U* test; *†* Chi-squared test

The operative approach was trans-thoracic in 233 (47.0%), trans-hiatal in 200 (40.3%), and a three-stage approach was used in six (1.2%) patients. Open and close laparotomy was performed in 57 (11.5%) patients either due to un-resectable tumor or metastatic disease. Pathological examination revealed R1 resection specimens (microscopic margin involvement) in 166 (33.5%) patients. Operative mortality occurred in 17 (3.4%) patients within 30 days of surgery. More trans-hiatal esophagectomies and three-stage esophagogastrectomies were performed in the post-PET/CT cohort than the pre-PET/CT cohort, which contained more trans-thoracic esophagectomies (*p* < 0.0001). The post-PET/CT cohort also underwent marginally more open and close laparotomies for undetected distant metastases (*n* = 27, 12.1%) compared with the pre-PET/CT cohort (*n* = 30, 11%, *p* = 0.698). The median follow-up was 26 months (range, 6 to 220), with 476 patients (96.1%) followed up for 1 year or until death, 447 (90.0%) for 2 years, 418 (84.3%) for 3 years, and 375 (81.4%) for 5 years.

### Duration of survival

#### Disease-free survival

**Fig. 3:**
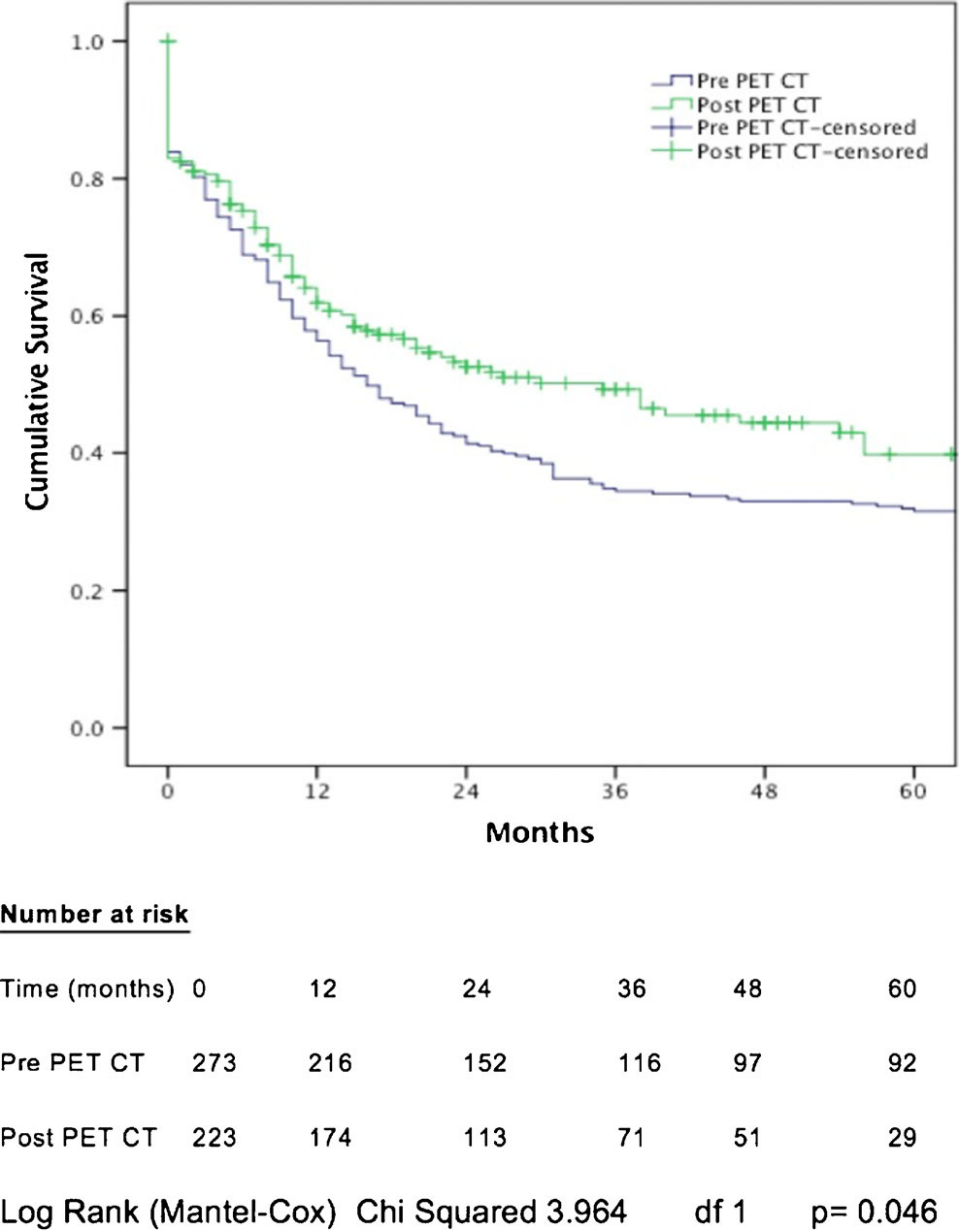
Cumulative disease-free survival related to introduction of PET/CT

For patients with all pathological stages of disease, median DFS was 16 months in the pre-PET/CT cohort, compared with 35 months in the post-PET/CT cohort (*p* = 0.049; Fig. 3). One-, 2-, and 3-year cumulative DFS in the pre-PET/CT cohort was 56.5, 41.4, and 34.4% respectively, compared with 61.9, 52.6, and 49.3%, in the post-PET/CT cohort. Following PET/CT introduction, median-, 1-, 2-, and 3-year DFS increased by 19 months, 5.4, 11.2, and 14.9%, respectively. All factors associated with DFS on univariable analysis are shown in Table 2.

**Table 2:** Univariable analysis of factors associated with disease-free survival

	Chi^2^	df	*p* value
Gender	0.446	1	0.504
Age	3.427	3	0.330
Tumor histology	0.164	1	0.686
PET/CT	3.964	1	**0.046**
PET/CT scanner	0.244	1	0.621
Neoadjuvant therapy (all types)	10.837	1	**0.001**
Neoadjuvant chemoRx	19.474	1	**< 0.0001**
Neoadjuvant CRTx	0.684	1	0.408
Surgery type*	2.620	2	0.270
pT stage	390.092	5	**< 0.0001**
pN stage	418.258	4	**< 0.0001**
R1 resection	365.946	2	**< 0.0001**

*df* degrees of freedom, *Age* < 50, 50–59, 60–79, > 70, *PET/CT location*, Cheltenham/Cardiff, *Neoadjuvant therapy* –all types, chemotherapy/chemo radiotherapy, *Neoadjuvant chemoRx* neoadjuvant chemotherapy, *Neoadjuvant CRTx*, neoadjuvant chemo radiotherapy, *Surgery type* *Trans hiatal esophagectomy/trans thoracic esophagectomy/3-stage esophagectomy, *pT* pathological T stage, *pN* pathological N stage, *R1* positive resection margin

#### Overall survival

**Fig. 4:**
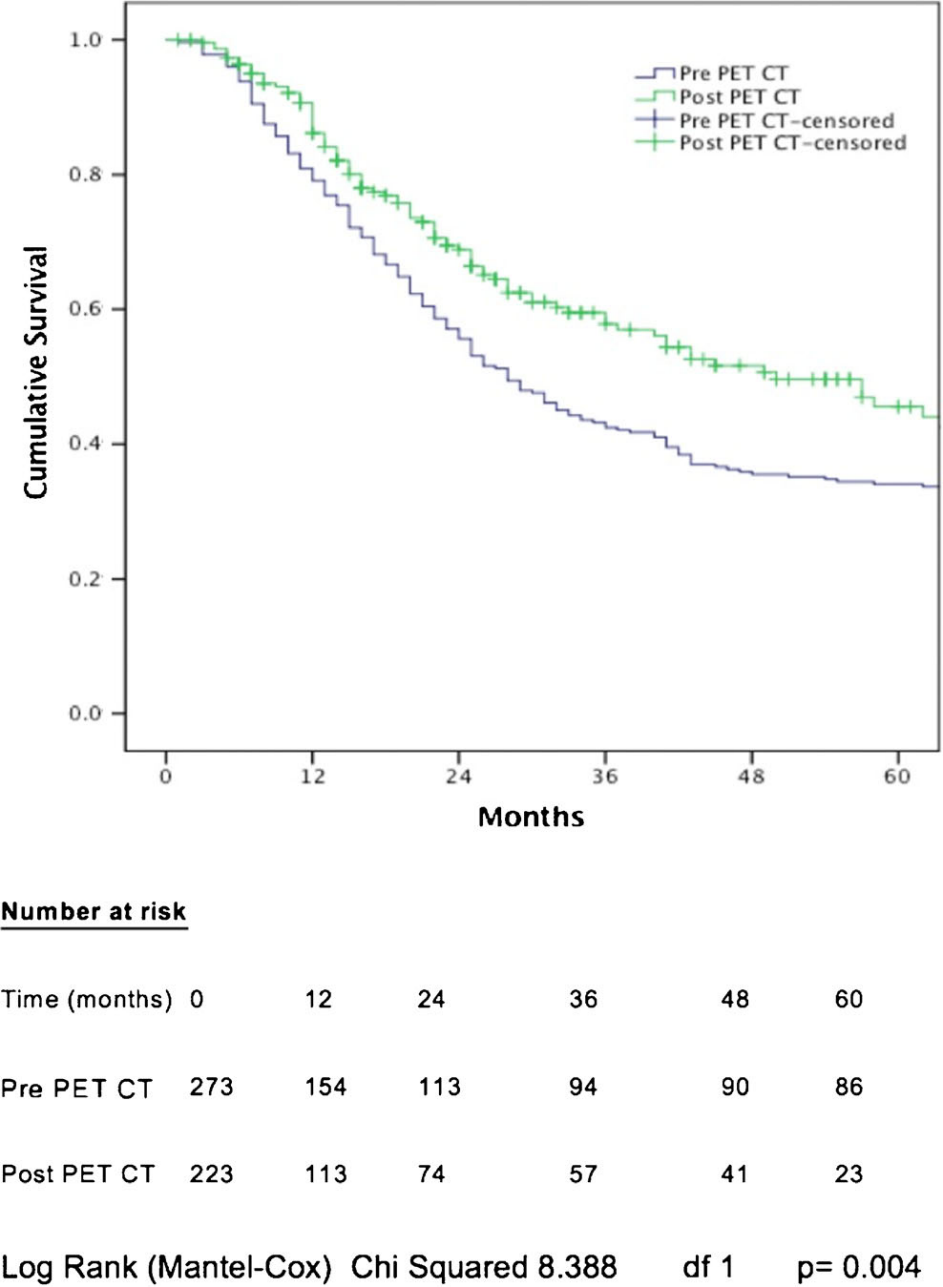
Cumulative overall survival related to introduction of PET/CT

For patients with all pathological stages of disease, median OS was 28 months in the pre-PET/CT cohort, compared with 50 months in the post-PET/CT cohort (*p* = 0.004; Fig. 4). One-, 2-, and 3-year cumulative OS in the pre-PET/CT cohort was 79.1, 55.7, and 42.5%, respectively, compared with 86.2, 68.8, and 57.8% in the post-PET/CT cohort. Following PET/CT introduction, the median-, 1-, 2-, and 3-year OS increased by 22 months, 7.1, 13.1, and 15.3%, respectively. All factors associated with OS on univariable analysis are shown in Table 3.

**Table 3:** Univariable analysis of factors associated with overall survival

	Chi^2^	df	p value
Gender	0.246	1	0.620
Age	4.609	3	0.203
Tumor histology	0.032	1	0.859
PET/CT	8.388	1	**0.004**
PET/CT scanner	0.306	2	0.580
Neoadjuvant therapy (all types)	10.837	1	**0.001**
Neoadjuvant chemoRx	17.388	1	**< 0.0001**
Neoadjuvant CRTx	1.028	1	0.311
Surgery type*	4.356	2	0.113
pT stage	160.877	5	**< 0.0001**
pN stage	177.154	4	**< 0.0001**
R1	136.000	2	**< 0.0001**

*df* degrees of freedom, *Age* < 50, 50–59, 60–79, > 70, *PET/CT location*, Cheltenham/Cardiff, *Neoadjuvant therapy* –all types, chemotherapy/chemo radiotherapy, *Neoadjuvant chemoRx* neoadjuvant chemotherapy, *Neoadjuvant CRTx*, neoadjuvant chemo radiotherapy, *Surgery type* *Trans hiatal esophagectomy/trans thoracic esophagectomy/3-stage esophagectomy, *pT* pathological T stage, *pN* pathological N stage, *R1* positive resection margin

#### Multivariable analysis

The factors found to be significantly associated with DFS and OS on univariable analysis were entered into a multivariable analysis using Cox’s proportional hazards model, the results of which are shown in Table 4. The number of events per variable was 38.14.

**Table 4:** Multivariable analysis of factors associated with disease-free and overall survival

Variable	HR	95% CI	*p* value
Disease-free survival			
pT stage	1.526	1.31–1.78	< 0.0001
pN stage	1.371	1.26–1.49	< 0.0001
Overall survival			
PET/CT introduction	0.688	0.53–0.89	0.004
pT stage	1.496	1.28–1.75	< 0.0001
pN stage	1.114	1.04–1.19	0.001

*pT* pathological T stage, *pN* pathological N stage

#### Recurrence rates

Table 5 illustrates the recurrence patterns within the two cohorts. The overall number of patients diagnosed with cancer recurrence was 199 (45.3%).

**Table 5:** Recurrence rates related to cohort for patients who received curative surgery

	Pre-PET/CT	Post-PET/CT	Total	*p* value
Number of patients*	243	196	439	
Recurrence	125 (51.4)	74 (37.8)	199	**0.004**
Site of recurrence				
Locoregional	55 (22.6)	34 (17.3)	89	0.171
Distant	96 (39.5)	53 (27.0)	149	**0.006**
Time to recurrence (months)				
Overall	15 (2–85)	10 (2–93)		0.308
Locoregional	15 (2–85)	14.5 (4–93)		0.392
Distant	14 (2–72)	10 (2–69)		0.7
Open and close laparotomy	30	27	57	

Figures are numbers of patients with percentages in parentheses; Site of recurrence, inclusive of patients diagnoses with both locoregional and distant disease at time of diagnosis;

*Excluding open and close procedures

Recurrent cancer was observed in 125 patients (51.4%) in the pre-PET/CT cohort, compared with 74 patients in the post-PET/CT cohort (37.8%, Chi^2^ 8.199; df 1, *p* = 0.004). The site of recurrent cancer was less likely to be distant in location after PET/CT introduction (39.5 vs. 27.0%, *p* = 0.006).

## Discussion

This is the first study and propensity score analysis to demonstrate a positive significant correlation between the introduction of PET/CT into an EC staging algorithm and a reduction in cancer recurrence, with a commensurate increase in durations of survival, irrespective of stage, in patients undergoing potentially curative surgery.

In keeping with previous reports [5, 6], PET/CT upstaged 78 patients (13.2%), changing their treatment modality and precluding surgery. Median-, 1-, and 3-year OS increased significantly by 22 months, 13.1 and 15.3%, respectively after introduction of PET/CT. Cancer recurrence was 13.6% less common after PET/CT introduction, with fewer loco-regional and distant recurrence events when compared with historical controls. Consequently, the hypothesis addressed, namely that introduction of PET/CT into the routine EC staging algorithm was associated with improved OS after potentially curative surgery, was proven correct.

Reports regarding the influence of PET/CT within EC staging algorithms on patient long-term outcomes related to recurrence and durations of survival are few. Torrance et al. [8], from Cheltenham, England, reported in a retrospective review of 200 EC patients undergoing PET/CT, 128 of who underwent esophageal resection. Although PET/CT altered treatment intent in 19 patients (9.5%), no significant difference was noted in post-operative mortality, or early recurrence where PET/CT was performed when adjusted for age, gender, stage, or neoadjuvant chemotherapy (odds ratio 1.136, *p* = 0.761). Moreover, PET/CT had no significant effect on survival (Chi^2^ 0.710, *p* = 0.400). The difference in survival pre- and post-PET/CT was approximately 2, 6, and 7% at 1, 2, and 3 years, compared with 7.1, 13.1, and 15.3% in this study. Torrance et al. concluded that PET/CT improved the accuracy of EC staging, avoiding potentially unnecessary surgery, and contended that missed occult metastases did not appear to be the primary cause of early EC recurrence.

The current study has potential inherent limitations. The PET/CT examinations were performed at two centers, using different scanners, protocols, and uptake times, with patients in the early part of the study referred to site 1 (Philips 16 slice Gemini GXL), prior to the opening of site 2’s PET/CT in 2010. The main differences between the two centers were firstly, a 60-min uptake time on the first scanner (site 1), and a 90-min uptake time in site 2. Longer uptake times lead to higher tumor-to-background tracer uptake and may therefore increase conspicuity of nodal and distant metastases [23]. Secondly, the site 2 scanner had time of flight correction whereas the site 1 scanner did not. Time-of-flight reconstructions improve signal-to-noise ratio and improve lesion conspicuity [24]. Thirdly, at the site 1 center, images were acquired for 4 min per bed position, whereas at site 2, the acquisition was 3 min per bed position. This would be expected to lead to some improvement of image quality at the site 1 center, provided that the patient was able to remain motionless throughout the longer acquisition, which might mitigate the other factors. The potential heterogeneity introduced within the PET/CT imaged group was a reason why the location of the PET/CT scan was a covariate within the propensity score and as a result, adds great strength to this study.

The study included patients treated over a 17-year period with a variety of treatment strategies. Evolution in UGI cancer practice would have naturally occurred during the time frame of this study, including neoadjuvant therapy regimen modifications, more detailed patient risk profile assessment related to patients’ fitness for surgery including objective assessment of physical fitness with cardiopulmonary exercise testing, centralization of surgical services, and the introduction of an Enhanced Recovery Program, all of which might represent potential confounding factors.

Conversely, the strengths of the study are that the data was collected prospectively, from a well-defined geographical area served by an established regional UGI cancer network and multidisciplinary team. This team included six experienced specialist surgeons, with a referring population base of 1.8 million, accepting over 500 cases per year, generating in excess of 100 potentially curative esophagogastric resections, whose outcome data is well audited and of public record [25]. The survival and prognostic data are especially robust because no patients were lost to follow-up and causes and dates of death were obtained from death certificates provided by the Office for National Statistics. Moreover, the improvement in survival cannot be explained by poor outcomes in the historical control cohort, which compare favorably with the clinical outcomes data published in the most recent NOGCA report [26]. NOGCA cumulative survival at 1, 2, and 3 years was approximately 70, 50, and 40% compared with 79.1, 55.7, and 42.5% in propensity matched historical controls. Furthermore, NOGCA reported 30-day mortality of 4.5%, between 2007 and 2009, comparable with the 4% observed in the propensity matched historical cohort of this study.

Multivariable model analyses have been considered the orthodox and preferred statistical way of assessing the effect of possible predictors on outcomes after controlling for baseline characteristics, yet their suitability depends on consistent assumptions underlying any given model. Regression analyses of DFS and OS by means of propensity scores overcome some potential biases, and although it is considered the most useful statistical method for controlling confounders, providing appropriate estimates even when faced with extreme correlation between confounders and the exposure [27], it cannot account for unknown confounders [28]. Propensity score analysis is well suited when several risk-adjusted outcomes are under assessment (DFS and OS), because such scoring simplifies multiple outcome weighting, which once calculated can be related to each specific outcome measure.

In conclusion, this propensity score regression analysis further supports the use of PET/CT in EC staging pathways, and in the absence of an adequately powered randomized controlled trial, provides the best level of research evidence regarding the influence of PET/CT on outcome after potentially curative esophagectomy for cancer. Risk profile assessment represents an important development in the selection algorithm for patients diagnosed with invasive EC. While this is often assumed to relate to patients’ physical fitness, clearly avoiding hopeless radical, and unnecessary surgery, in patients with undetected occult metastases is an important allied strategy, if EC treatment outcomes are to be optimized. The findings of this study have shown that the introduction of PET/CT into the global patient assessment process changed the risk profile of over one in ten patients, reduced global recurrence by one-quarter, and improved median survival by a full year. A health economic analysis of the cost-effectiveness of PET/CT is now desirable to estimate the potential allied clinical and fiscal benefits available to upper gastrointestinal cancer networks.
